# Electroacupuncture-Induced Cholinergic Nerve Activation Enhances the Hypoglycemic Effect of Exogenous Insulin in a Rat Model of Streptozotocin-Induced Diabetes

**DOI:** 10.1155/2011/947138

**Published:** 2011-06-27

**Authors:** Yu-Chen Lee, Te-Mao Li, Chung-Yuh Tzeng, Yu-Wen Cheng, Ying-I Chen, Wai-Jane Ho, Jaung Geng Lin, Shih-Liang Chang

**Affiliations:** ^1^Department of Acupuncture, China Medical University Hospital, Taichung, Taiwan; ^2^School of Chinese Medicine, China Medical University, Taichung, Taiwan; ^3^College of Life Sciences, National Tsing Hua University, Hsinchu, Taiwan; ^4^Department of Orthopedics, Taichung Veterans General Hospital, Taichung, Taiwan; ^5^Department of Internal Medicine, Lee's General Hospital, Miaoli, Taiwan; ^6^Department of Medicinal Botanicals and Health Care, Da-Yeh University, Chunghwa, Taiwan; ^7^College of Life Sciences, National Chung Hsing University, Taichung, Taiwan

## Abstract

The aim of this study is to explore the mechanisms by which electroacupuncture (EA) enhances the hypoglycemic effect of exogenous insulin in a streptozotocin- (STZ-) diabetic rats. Animals in the EA group were anesthetized and subjected to the insulin challenge test (ICT) and EA for 60 minutes. In the control group, rats were subjected to the same treatment with the exception of EA stimulation. Blood samples were drawn to measure changes in plasma glucose, free fatty acids (FFA), and insulin levels. Western blot was used to assay proteins involved in insulin signaling. Furthermore, atropine, hemicholinium-3 (HC-3), and Eserine were used to explore the relationship between EA and cholinergic nerve activation during ICT. EA augmented the blood glucose-lowering effects of EA by activating the cholinergic nerves in STZ rats that had been exposed to exogenous insulin. This phenomenon may be related to enhancement of insulin signaling rather than to changes in FFA concentration.

## 1. Introduction

Insulin therapy has revolutionized the treatment of diabetes mellitus (DM) and has contributed to increased longevity and improved quality of life for people with diabetes [1]. Insulin resistance, however, can develop up to a decade before the onset of diabetes, and insulin insensitivity increases the risk of macroangiopathy [2]. According to Zimmet et al., the worldwide prevalence of diabetes will increase by 50% by 2010, with the highest increases occurring in Asian countries [3]. In order to prevent the formation of insulin resistance, agents and/or methods for enhancing insulin sensitivity become important topic recently.

Previous research on the use of electroacupuncture (EA) to reduce blood glucose levels showed that using specific frequencies to stimulate points on the middle part of the abdomen (Zhongwan acupoint) of rats could significantly reduce plasma glucose levels. The mechanism of action involved the stimulation of the release of *β*-endorphins, which subsequently increased insulin production in animal models of type II DM. In addition, stimulation of Zhongwan acupoints results in significantly greater plasma glucose-lowering effects than stimulation of adjacent nonacupuncture points [4]. Other studies showed that EA applied to the Zusanli (ST-36) or Zhongwan acupoints enhanced insulin sensitivity in rats although the mechanisms of action were not investigated [5, 6].

In the selection of parameters and acupoints for EA to lower plasma glucose, previous experiments performed on normal Wistar rats compared the glucose-lowering effects of different EA frequencies (2 Hz, 15 Hz, and 100 Hz) applied to Zhongwan acupoints. The most significant reduction in plasma glucose levels was elicited by EA at a frequency of 15 Hz [4]. Chang et al. compared the plasma glucose-lowering effects of EA (2 Hz) applied to acupoints on the foot (Zusanli acupoint) and on the abdomen of rats (Zhongwan acupoint). The results showed that stimulation of the Zusanli acupoint had better glucose-lowering effects than stimulation of the Zhongwan acupoint. The glucose-lowering effects of EA applied to the Zusanli acupoint involve the release of serotonin in addition to endogenous opioid peptides [7]. The results from those studies imply that stimulation of the Zusanli acupoint elicits a better hypoglycemic effect than stimulation of the Zhongwan acupoint.

Otherwise, Shapira et al. applied EA to the Zhongwan acupoint of fat sand rats with insulin resistance and found that EA produced a sustained noninsulin dependent reduction in glucose levels. They reported that their protocol effectively delayed disease onset in animals and increased the survival rate [8]. Chang et al. used the insulin challenge test (ICT) and intravenous glucose tolerance test (IVGTT) to study whether 15 Hz stimulation of Zusanli acupoints increased insulin sensitivity [5]. They found that the effect was even more apparent in animals that had been injected with steroids to induce insulin resistance and that the sensitizing effects were related to free fatty acids (FFAs) in steroid background rats (SBRs) [9, 10]. FFAs cause insulin resistance in all major insulin target organs (skeletal muscle, liver, and endothelial cells) and have emerged as a major link between obesity, the development of the metabolic syndrome, and atherosclerotic vascular disease [11]. The homeostatic model assessment index (HOMA) is an easy way to evaluate insulin resistance [10, 12]; therefore, in this study, the FFAs and HOMA were used to explore the effects of EA on enhancing the hypoglycemic effect of exogenous insulin.

A recent study showed that EA at the Zusanli acupoint reduced plasma glucose levels by stimulating the cholinergic nerves and by inducing the upregulation of the insulin signaling proteins IRS-1 and AKT-2 [13]. Hsieh et al. tested the effects of low-frequency (2 Hz) and high-frequency (100 Hz) EA stimulation at the ST-36 acupoint on heart rate and skin temperature in humans and found that high-frequency EA stimulation led to a reduction in heart rate by activating parasympathetic nerves [14]. Thus, we hypothesized that the cholinergic nerves are involved in enhancing exogenous insulin sensitivity in a STZ-induced diabetic rat model. The aim of this study was to explore the mechanisms by which EA enhances the hypoglycemic effect of exogenous insulin in a streptozotocin- (STZ-) induced diabetic rat model. We used antagonists of cholinergic nerves, Atropine, Hemicholinium-3 (HC-3), and Eserine, to explore the relationship between cholinergic nerve stimulation and EA-induced exogenous insulin sensitivity. Atropine is a competitive antagonist of the muscarinic acetylcholine receptor; HC-3 indirectly reduces Ach by suppressing choline reuptake in the cholinergic nerve ending, thereby reducing Ach concentrations between synapses and suppressing the activity of cholinergic nerves [13, 15, 16], and Eserine is an inhibitor of cholinesterase for enhancing cholinergic tone [17]. They were used through three different ways for exploring the mechanisms relative to cholinergic nerve in EA actions.

## 2. Materials and Methods

### 2.1. Animal Model

Normal male Wistar rats weighing approximately 250–350 g and aged 8–10 weeks were purchased from the BioLASCO animal center. Type 1 diabetes was induced by administration of STZ (60 mg/kg, i.v.) via the femoral vein under a fasting state [13]. Animals were housed in plexiglass cages at a constant room temperature of 22 ± 2°C with a relative humidity of 65 ± 5%. Rats were fed standard rat chow and were given free access to water. Animals were randomly divided into experimental groups and control groups after an adaptation period of one week, and all the animals in this study were anesthetized using pentobarbital (40 mg/kg i.p.). All animals were treated in accordance with the National Institute of Health (NIH) Guide for the Care and Use of Laboratory Animals, and the study protocol was approved by the ethics committee of the China Medical University, Taichung, Taiwan.

### 2.2. Electroacupuncture (EA)

Acupoints were located according to body length measurement as described elsewhere [18]. The ST-36 acupoint was located on the anterior tibia muscle approximately 5 mm below the knee. Bilateral ST-36 acupoints were punctured in a vertical and deep manner with 1.27 cm, 32 gauge acupuncture needles. After a 5-min needling period, EA was performed for 30 minutes at a frequency of 15 Hz and the amplitude of 10 mA using a HANS LY257 acupoint and nerve stimulator (Healthtronics, Singapore).

### 2.3. Plasma Glucose and FFA Assay

Approximately 0.3–0.5 mL of blood was obtained from a femoral vein under anesthesia using a 1 mL syringe containing heparin. The collected blood was introduced into eppendorff tubes, lightly shaken, and then stored on ice. Following centrifugation at 21380 ×g for 50 minutes, the plasma glucose (mg/dL) and plasma FFA levels (meq/L) were determined using a spectrophotometer (COBAS^R^ system, Roche, Switzerland, USA).

### 2.4. Insulin Challenge Test and Plasma Insulin Assay

After administration of regular insulin 1 U/kg i.p. (Novo Nordisk Company, Denmark) 30 min after onset of hypoglycemia [5], blood samples were taken from a femoral vein at 0, 30, and 60 min and assayed for plasma glucose levels. Plasma insulin levels were measured using an ELISA kit (EZRMI-13K, Linco Research, Inc., USA). In brief, samples were incubated for 2 h at room temperature in a shaker and then exposed to peroxidase conjugate and antibodies bound to a microtitration well. A conjugate was detected by reaction with 3,3′,5,5′-tetramethylbenzidine (TMB). The reaction was stopped by adding acid. The mixture was shaken to produce a colorimetric endpoint that was read using a spectrophotometer. The values obtained were noted as pmol of peptide per liter of plasma.

### 2.5. Study Protocol

#### 2.5.1. Plasma Glucose Reduction under Insulin Challenge

STZ-induced diabetic rats (*N* = 19) were randomly divided into an EA group (*N* = 8) or a control group (*N* = 11). Animals in the EA group were anesthetized, subjected to the ICT, and then stimulated by EA for 60 minutes. Rats in the control group were anesthetized and subjected to ICT but did not undergo EA. Blood was extracted for glucose, FFAs, and insulin testing at the beginning of the experiment, 30 minutes of the experiment, and 60 minutes of the experiment as previously described [5].

#### 2.5.2. Pharmacological Exploration of Mechanisms

**Figure 1:**
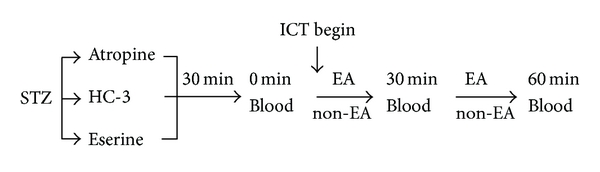
Time schedule of the protocol for exploring the relationship between the cholinergic nerve and the enhancing hypoglycemic effect of insulin by EA: STZ: streptozotocin-induced diabetic rats, ICT begin: the beginning point of insulin challenge test, Atropine/HC-3/Eserine: each agent was given at 30 min before ICT separately, EA: the experimental group treated EA, non-EA: the control group not treated EA.

The pharmacological agents, Atropine 0.1 mg/kg, HC-3 0.01 mg/kg, and Eserine 0.01 mg/kg, were injected separately into the abdomens of STZ-induced diabetic rats 30 minutes prior to the experiment in order to block or enhance cholinergic activity [13, 17]. A total of 48 rats were used to explore the mechanism. After exposure to the three pharmacological agents, rats (*N* = 16) were then randomly divided into an EA group (*N* = 8) or a control group (*N* = 8). Animals in the EA group were subjected to the ICT and then EA stimulation for 60 minutes. Rats in the control group were anesthetized and subjected to ICT but did not receive EA. Blood was extracted for glucose testing prior to the experiment and at 30 and 60 minutes into the experiment as previously described [5]. The protocol of this procedure is summarized in Figure 1, and the two groups (EA and non-EA) were compared in the same background of cholinergic tone [19]. In the atropine blocking experiment, the plasma insulin and FFAs were also assayed.

#### 2.5.3. Western Blotting Analysis

Fasting STZ rats (*N* = 12) were randomly divided into EA and non-EA groups. The other fasting STZ rats (*N* = 12) were randomly divided into an EA and non-EA group 30 min after treatment with atropine 0.1 mg/kg, i.p. At the end of EA treatment (30 min) in each group, portions of the gastrocnemius muscles were taken as samples for analysis of insulin signaling proteins (IRS-1, AKT-2). Muscle samples were homogenized in buffer solution before centrifugation at 13980 ×g. The obtained supernatant was used to estimate the amount of protein using an assay kit from Bio-Rad Laboratories. The supernatant (protein) was mixed with 4X SDS-loading dye and boiled for 15 min at 95°C for denaturing. Separating (8%) and stacking gels were prepared. Then, protein (90 *μ*g/mL) in buffer was loaded into each well for electrophoresis. Proteins were electrophoretically transferred to polyvinylidene difluoride (PVDF) membranes at 4°C. The membranes were then blocked with 5% nonfat dry milk in phosphate-buffered saline (PBS) for 1 hr at room temperature and incubated with the specific primary antibodies (Santa Cruz Biotechnology, Inc.). After the membranes had been washed in a buffer containing 0.1% Tween 20 in 1 × PBS, blots were incubated with a horseradish peroxidase-linked specific secondary antibody (Santa Cruz Biotechnology, Inc.) and then evaluated using an enhanced chemiluminescence detection using ECL reagent plus (PerkinElmer Life Sciences, Inc.). Band intensities were quantified by densitometry to observe the target proteins.

### 2.6. Statistical Analysis

All values are expressed as mean ± SE. The HOMA was used to evaluate insulin resistance. HOMA = insulin (*μ*U/mL) × glucose (mmol/L)/22.5 [10, 12]. The parameter, area under curve (AUC), is calculated for comparison by the statistical software, MedCalc. Differences in mean values between the experimental group (EA) and control group (non-EA) were tested by the Student's *t*-test. A self-paired *t*-test was used to determine significant changes in levels of plasma indicators before and after treatment. For all comparisons, a *P* value <.05 (two-sided) was considered to represent statistical significance.

## 3. Results

### 3.1. Effects of EA on STZ Rats under ICT

**Figure 2:**
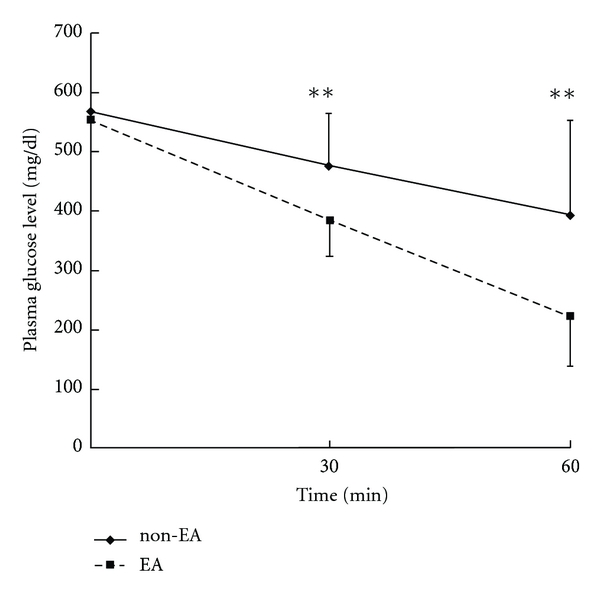
The plasma glucose-level-lowering effect of EA applied to the bilateral Zusanli acupoints on STZ rats after ICT. EA represents 12 hours of fasting and application of EA; non-EA shows the same except application of EA. Comparison of plasma glucose levels 30 and 60 minutes between EA and non-EA group under ICT, **P < .01 by Student's *t*-test.

STZ-induced diabetic rats (*N* = 19) were first subjected to ICT and then randomly and equally divided into an EA group or a non-EA group. Rats in the EA group received EA at the Zusanli acupoint for 60 minutes. Rats in the control group did not receive EA. After 60 minutes, the mean blood glucose level in the EA group was 224.0 ± 86.2 mg/dL and that in the control group was 393.6 ± 160.2 mg/dL. Also, the AUC of EA group was 23202 ± 3411 and that in the non-EA group was 28737 ± 6181. The differences of plasma glucose level after 60 min and AUC between EA and non-EA were significant (*P* < .05) (Figure 2).

### 3.2. Blockage Effects of Atropine on STZ Rats Subjected to ICT

**Figure 3:**
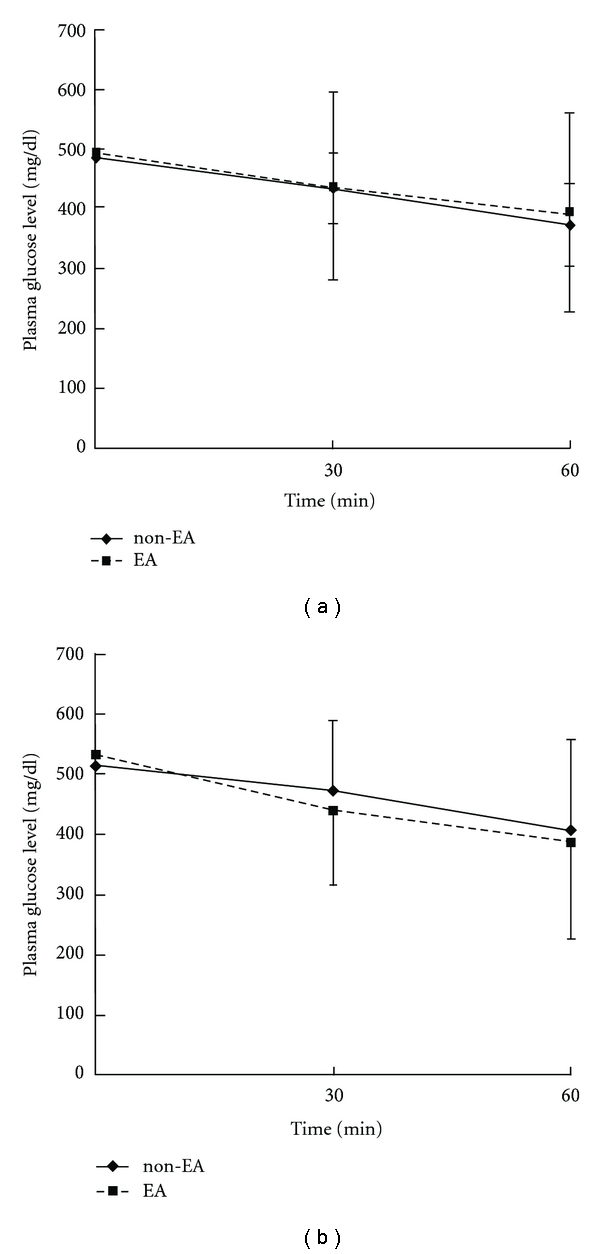
(a) Blocking effect of atropine on STZ rats received EA during ICT (b) Impact of HC-3 on the plasma glucose-lowering action in STZ rats subjected to ICT. EA represents 12 hours of fasting and application of EA; non-EA shows the same without application of EA. The plasma glucose levels were compared between EA and non-EA group at 30 and 60 minutes during ICT by Student's *t*-test.

After administering Atropine 0.1 mg/kg i.p., 15 Hz EA was applied to the Zusanli acupoint in normal rats following ICT. The blood glucose levels were then compared with those in the non-EA group. There were no significant differences in blood glucose levels between the two groups. After 60 minutes, the mean blood glucose level in the EA group was 394.5 ± 167.0 mg/dL and that in the control group was 373.6 ± 67.8 mg/dL. Also, the AUC of EA group was 26502 ± 9264 and that in the non-EA group was 25896 ± 3795. The differences of plasma glucose level after 60 min and AUC between EA and non-EA were not significant (*P* > .05), indicating that atropine effectively blocked the glucose-lowering effects of EA in STZ-induced diabetic rats that had been subjected to insulin challenge (Figure 3(a)).

### 3.3. Blockage Effects of HC-3 on STZ Rats Subjected to ICT

After administering HC-3 0.01 mg/kg i.p., 15 Hz EA was applied to the Zusanli acupoint in STZ-induced diabetic rats under ICT. The blood glucose values were compared with those in the non-EA group. No significant differences in blood glucose levels were noted. After 60 minutes, the mean blood glucose level in the EA group was 385.5 ± 161.4 mg/dL and that in the non-EA group was 406.7 ± 149.7 mg/dL. Also, the AUC of EA group was 26911 ± 7742 and that in the non-EA group was 27985 ± 7709. The differences of plasma glucose level after 60 min and AUC between EA and non-EA were not significant (*P* > .05), indicating that HC-3 effectively blocked the glucose-lowering effects of EA in STZ-induced diabetic rats that had been subjected to insulin challenge (Figure 3(b)).

### 3.4. Effects of Eserine on STZ Rats Subjected to ICT

**Figure 4:**
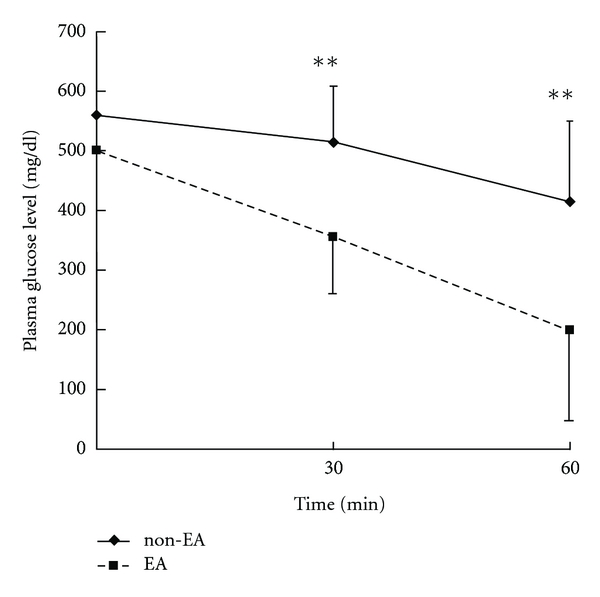
Effect of Eserine on the plasma glucose reduction effects of EA applied to the Zusanli acupoint in STZ rats during ICT. EA represents 12 hours of fasting and application of EA; non-EA shows the same without application of EA. The plasma glucose levels were compared between EA and non-EA group at 30 and 60 minutes during ICT, **P < .01 by Student's *t*-test.

After administering Eserine 0.01 mg/kg i.p., 15 Hz EA was applied to the Zusanli acupoint in STZ-induced diabetic rats after ICT. The blood glucose values were compared with those in the non-EA group. The results showed that 30 minutes after rats had been injected with Eserine (0.01 mg/kg i.p.), EA (15 Hz) applied to the Zusanli acupoint for 60 min led to a 62.1% reduction (from 500.6 ± 57.6 to 199.8 ± 151.8 mg/dL) in plasma glucose. This represents a significant increase over the 26.6% (from 560.9 ± 69.9 to 415.6 ± 136.1 mg/dL) blood sugar reduction (non-EA) associated with EA plus Eserine (*N* = 8, *P* < .05) (Figure 4). Also, the AUC of EA group was 21203 ± 6226 and that in the non-EA group was 30114 ± 5906. The differences of plasma glucose level after 60 min and AUC between EA and non-EA were significant (*P* < .05), indicating that Eserine effectively augmented the blood glucose-lowering effects of EA in STZ rats that had been subjected to insulin challenge.

### 3.5. Changes in Insulin and FFA Concentrations and HOMA Values under ICT

EA (15 Hz) was applied to the Zusanli acupoint in STZ rats under ICT. The insulin and FFA values were then compared with those in the non-EA group. No significant differences in insulin or FFA values were noted between the two groups; however, HOMA values were significantly lower in the EA group than in the non-EA group (**P < .05) (Table 1). Changes in HOMA values were attributed to reductions in insulin and not to FFA concentrations (Table 1).

### 3.6. Impact of Atropine on Changes in Insulin, FFA, and HOMA Values under ICT

Insulin and FFA values in the EA group after ICT challenge and application of EA (15 Hz) were compared with those in the non-EA group under ICT. The *t*-test revealed no significant differences in insulin, FFA, or HOMA values between the two groups (*N* = 8) (Table 2).

### 3.7. The Effect of EA on Expression of Insulin Signaling Proteins

**Figure 5:**
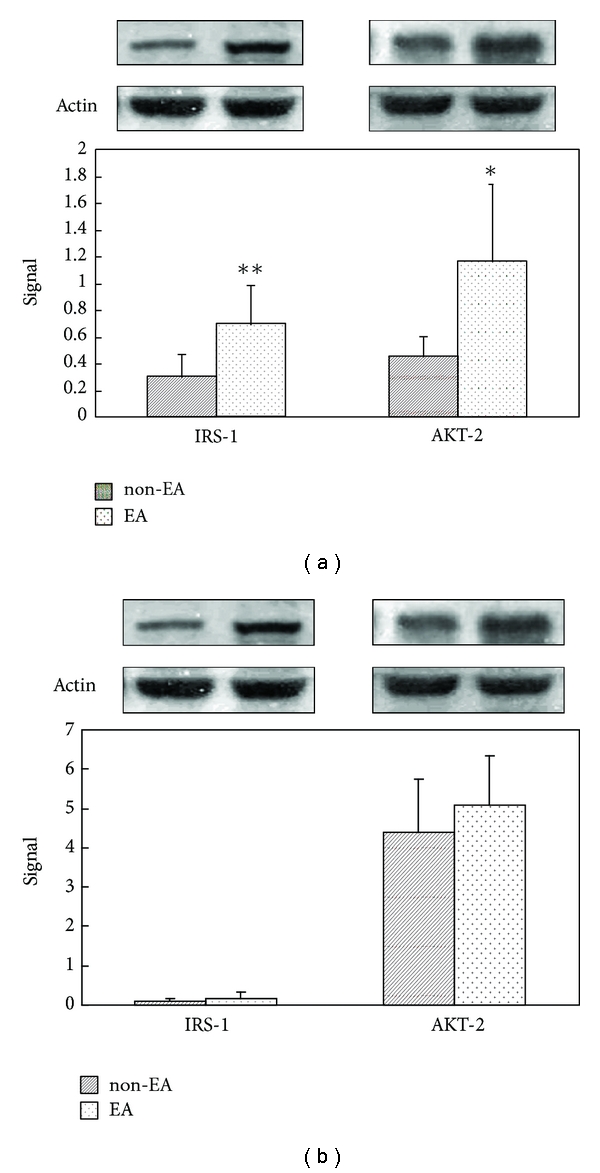
(a) The EA-induced change of insulin signals. (b) The blocking effect of atropine on EA-induced change of insulin signals in STZ rats during ICT. The IRS-1 and AKT-2 ratios to actin were compared between EA and non-EA group by Student's *t*-test, *P < .05, **P < .01.

EA (15 Hz) was applied to the Zusanli acupoint in STZ rats under ICT. The levels of IRS-1 and AKT-2 protein expression were then compared between the EA group and the non-EA group. Western blot analysis revealed that the levels of IRS-1 and AKT-2 protein expression were significantly higher in rats that received EA than in rats that did not undergo EA stimulation (Figure 5(a)).

### 3.8. Atropine Blocks the Effect of EA in Expression of Insulin Signaling Proteins

After administering Atropine 0.1 mg/kg i.p., EA (15 Hz) was applied to the Zusanli acupoint in STZ rats under ICT. The levels of IRS-1 and AKT-2 protein expression were then compared between the EA group and the non-EA group. Western blot analysis revealed that the levels of IRS-1 and AKT-2 protein expression did not differ significantly between rats that received EA and rats that did not undergo EA stimulation (Figure 5(b)).

## 4. Discussion

This study shows that EA (15 Hz) augmented the glucose-lowering effects of exogenous insulin in STZ-induced diabetic rats (Figure 2). A previous study showed that the blood glucose-lowering effects of EA are related to the activation of cholinergic nerves and the adrenal gland in STZ rats [13]. In this study, Atropine, a cholinergic nerve antagonist, blocked the glucose-lowering effect of exogenous insulin in STZ-induced diabetic rats (Figure 3(a)). The results of this study also show that the blood glucose-lowering effects of 15 Hz EA in STZ-diabetic rats that had been subjected to ICT can be blocked by HC-3 (0.01 mg/kg i.p.) (Figure 3(b)). These results imply that secretion of acetylcholine (Ach) by cholinergic nerves augments the glucose-lowering effects of exogenous insulin in STZ rats.

Furthermore, Eserine, a drug that suppresses acetyl cholinesterase (AchE), causes Ach to accumulate at cholinergic synapses [13, 16, 17]. After a 30 minute exposure to Eserine (0.01 mg/kg i.p.), we found that the levels of plasma glucose were markedly lower in STZ-induced diabetic rats that had received EA (15 Hz) stimulation at the Zusanli acupoints than in rats that did not receive EA stimulation (*P* < .01, Figure 4), indicating that the cholinergic nerves involve in the increase of the hypoglycemic effect of exogenous insulin by EA.

In order to examine the effect of EA on exogenous insulin sensitivity, blood insulin and FFA levels were tested. Since the insulin and FFA levels did not change, and the HOMA decreased significantly after EA in STZ rats during ICT, we hypothesized that the EA-induced reduction in glucose was related to reduction in insulin resistance (Table 1). Furthermore, Atropine blocked the reduction in the HOMA after EA treatment. These results indicate that the hypoglycemic effect of EA is associated with cholinergic tone. Otherwise, FFA is the important factor in insulin resistance but it can be ruled out according to the no change by EA shown in Tables 1 and 2.

A recent study showed that EA offers a beneficial effect on insulin resistance in obese and diabetic db/db mice, at least partly, via stimulation of SIRT1/PGC-1*α*, thus resulting in improved insulin signaling [20]. Also, in rats with dihydrotestosterone- (DHT-)induced Polycystic ovary syndrome (PCOS), low-frequency EA has been shown to have systemic and local effects involving intracellular signaling pathways in muscle that may, at least in part, account for the marked improvement in insulin sensitivity [21]. In this study, Western blot was used to examine the expression of insulin signaling proteins in STZ rats under ICT. The results showed that IRS-1 and AKT-2 protein expression increased significantly (Figure 5(a)). Also, Atropine was able to block this message transmission pathway (Figure 5(b)). The results indicate that the activation of insulin signaling proteins, at least in part, is related to the cholinergic nerves. This upregulation of insulin signaling proteins was also obtained in a STZ-induced diabetic rat [13] and in a steroid-induced insulin resistance rat model [10]. This is the first time to report that the upregulation of insulin signaling proteins (IRS-1 and AKT-2) was obtained in a STZ-induced diabetic rat under ICT by EA. The phosphorylation of these signal proteins and further influence of insulin signal pathway deserve further research.

These results suggest that EA augments the blood glucose-lowering effect of EA in STZ rats that have been subjected to insulin challenge through the activation of the cholinergic nerves. This may be related to enhancement of insulin signaling rather than to change in insulin and FFA concentration.

## Figures and Tables

**Table 1:** EA (15 Hz) was applied to the bilateral Zusanli acupoints on STZ rats under ICT.

Plasma levels	Groups	0 min	30 min	60 min
Insulin (ng/mL)	EA	0.44 ± 0.30	3.66 ± 3.96	5.98 ± 2.42
	non-EA	0.33 ± 0.31	6.93 ± 4.17	5.94 ± 3.70

HOMA	EA	15.4 ± 11.2	77.9 ± 73.7*	65.6 ± 35.7*
	non-EA	10.2 ± 11.4	228.7 ± 92.5	152.4 ± 76.0

FFAs (mmol/L)	EA	1.05 ± 0.38	0.48 ± 0.31	0.44 ± 0.24
	non-EA	1.04 ± 0.33	0.47 ± 0.16	0.39 ± 0.19

All values are expressed as mean ± SE; HOMA = insulin (*μ*U/mL) × glucose (mmol/L)/22.5. The value of EA group (*N* = 8) was compared with the value of non-EA (*N* = 11) group by Student's *t*-test, *P < .05.

**Table 2:** Atropine blocking effect in 15 Hz EA on the bilateral Zusanli acupoints under ICT.

Plasma levels	Groups	0 min	30 min	60 min
Insulin (ng/mL)	EA	0.10 ± 0.09	1.97 ± 3.04	1.85 ± 2.89
	non-EA	0.04 ± 0.04	4.85 ± 6.97	4.10 ± 6.16

HOMA	EA	2.6 ± 3.2	73.6 ± 87.8	55.2 ± 69.4
	non-EA	1.2 ± 1.2	114.1 ± 162.0	81.6 ± 123.6

FFAs (mmol/L)	EA	0.97 ± 0.21	0.57 ± 0.27	0.57 ± 0.28
	non-EA	0.94 ± 0.42	0.62 ± 0.40	0.59 ± 0.46

All values are expressed as mean ± SE; HOMA = insulin (*μ*U/mL) × glucose (mmol/L)/22.5. The value of EA group (*N* = 8) was compared with the value of non-EA group (*N* = 8) by Student's *t*-test.

## Abbreviations

EA: Electroacupuncture
SBRs: Steroid background rats
STZ: Streptozotocin
ICT: Insulin challenge test
IVGTT: Intravenous glucose tolerance test
HC-3: Hemicholinium-3
FFAs: Free fatty acids
HOMA: Homeostatic model assessment index.

## References

[1] Lavernia F (2008). Treating hyperglycemia and diabetes with insulin therapy: transition from inpatient to outpatient care. *MedGenMed Medscape General Medicine*.

[2] Kuziemski K, Górska L, Jassem E, Madej-Dmochowska A (2009). Lung microangiopathy in diabetes. *Pneumonologia i Alergologia Polska*.

[3] Zimmet P, Alberti KGMM, Shaw J (2001). Global and societal implications of the diabetes epidemic. *Nature*.

[4] Chang SL, Lin JG, Chi TC, Liu IM, Cheng JT (1999). An insulin-dependent hypoglycaemia induced by electroacupuncture at the Zhongwan (CV12) acupoint in diabetic rats. *Diabetologia*.

[5] Chang SL, Lin KJ, Lin RT, Hung PH, Lin JG, Cheng JT (2006). Enhanced insulin sensitivity using electroacupuncture on bilateral Zusanli acupoints (ST 36) in rats. *Life Sciences*.

[6] Ishizaki N, Okushi N, Yano T, Yamamura Y (2009). Improvement in glucose tolerance as a result of enhanced insulin sensitivity during electroacupuncture in spontaneously diabetic Goto-Kakizaki rats. *Metabolism: Clinical and Experimental*.

[7] Chang SL, Tsai CC, Lin JG, Hsieh CL, Lin RT, Cheng JT (2005). Involvement of serotonin in the hypoglycemic response to 2 Hz electroacupuncture of zusanli acupoint (ST36) in rats. *Neuroscience Letters*.

[8] Shapira MY, Appelbaum EY, Hirshberg B, Mizrahi Y, Bar-On H, Ziv E (2000). A sustained, non-insulin related, hypoglycaemic effect of electroacupuncture in diabetic Psammomys obesus. *Diabetologia*.

[9] Cheng YW (2004). *Mechanisms of improving insulin resistance by electroacupuncture*.

[10] Lin RT, Tzeng CY, Lee YC (2009). Acute effect of electroacupuncture at the Zusanli acupoints on decreasing insulin resistance as shown by lowering plasma free fatty acid levels in steroid-background male rats. *BMC Complementary and Alternative Medicine*.

[11] Boden G (2008). Obesity and free fatty acids. *Endocrinology and Metabolism Clinics of North America*.

[12] Tremblay AJ, Lamarche B, Deacon CF, Weisnagel SJ, Couture P (2011). Effect of sitagliptin therapy on postprandial lipoprotein levels in patients with type 2 diabetes. *Diabetes, Obesity and Metabolism*.

[13] Lee YC, Li TM, Tzeng CY (2011). Electroacupuncture at the Zusanli (ST-36) acupoint induces a hypoglycemic effect by stimulating the cholinergic nerve in a rat model of streptozotocine-induced insulin-dependent diabetes mellitus. *Evidence-Based Complementary and Alternative Medicine*.

[14] Hsieh CL, Lin JG, Li TC, Chang QY (1999). Changes of pulse rate and skin temperature evoked by electroacupuncture stimulation with different frequency on both zusanli acupoints in humans. *American Journal of Chinese Medicine*.

[15] Zhao X (1995). Effect of HC-3 on electroacupuncture-induced immunoregulation. *Zhen Ci Yan Jiu*.

[16] Wu HT, Chang CK, Cheng KC, Chang CH, Yeh CH, Cheng JT (2010). Increase of plasma insulin by racecadotril, an inhibitor of enkephalinase, in wistar rats. *Hormone and Metabolic Research*.

[17] Liu KY, Wu YC, Liu IM, Yu WC, Cheng JT (2008). Release of acetylcholine by syringin, an active principle of Eleutherococcus senticosus, to raise insulin secretion in Wistar rats. *Neuroscience Letters*.

[18] Romita VV, Yashpal K, Hui-Chan CWY, Henry JL (1997). Intense peripheral electrical stimulation evokes brief and persistent inhibition of the nociceptive tail withdrawal reflex in the rat. *Brain Research*.

[19] Lin R-T, Chen C-Y, Tzeng C-Y (2011). Electroacupuncture improves glucose tolerance through cholinergic nerve and nitric oxide synthase effects in rats. *Neuroscience Letters*.

[20] Liang F, Chen R, Nakagawa A (2011). Low-frequency electroacupuncture improves insulin sensitivity in obese diabetic mice through activation of SIRT1/PGC-1*α* in skeletal muscle. *Evidence-Based Complementary and Alternative Medicine*.

[21] Johansson J, Feng Y, Shao R, Lönn M, Billig H, Stener-Victorin E (2010). Intense electroacupuncture normalizes insulin sensitivity, increases muscle GLUT4 content, and improves lipid profile in a rat model of polycystic ovary syndrome. *American Journal of Physiology*.

